# Risk of venous thromboembolism in people with RA: a population-based study in the UK

**DOI:** 10.1093/rheumatology/keaf430

**Published:** 2025-08-07

**Authors:** Mark D Russell, Katie Bechman, Mark Gibson, Victoria Basey, Michael Mclean, Saqib Rana, Anna Barkaway, Simon de Lusignan, Maya H Buch, James B Galloway

**Affiliations:** Centre for Rheumatic Diseases, King’s College London, London, UK; Centre for Rheumatic Diseases, King’s College London, London, UK; Centre for Rheumatic Diseases, King’s College London, London, UK; Pfizer Ltd, Tadworth, UK; Pfizer Ltd, Tadworth, UK; Pfizer Ltd, Tadworth, UK; Pfizer Ltd, Tadworth, UK; Nuffield Department of Primary Care Health Sciences, University of Oxford, Oxford, UK; Royal College of General Practitioners Research and Surveillance Centre (RSC), London, UK; Centre for Musculoskeletal Research, Division of Musculoskeletal & Dermatological Sciences, Faculty of Biology, Medicine & Health, University of Manchester, Manchester, UK; NIHR Manchester Biomedical Research Centre, Manchester, UK; Centre for Rheumatic Diseases, King’s College London, London, UK

**Keywords:** RA, venous thromboembolism, pulmonary embolism, safety, epidemiology, obesity

## Abstract

**Objectives:**

To evaluate the absolute and relative risk of venous thromboembolism (VTE) in individuals with RA, with relation to age, sex, BMI, disease duration and exposure to exogenous oestrogens.

**Methods:**

Individuals with RA, registered with the UK Oxford-RCGP RSC primary care database between 1999 and 2018, were matched 1:4 with individuals without RA. Multivariable-adjusted Cox proportional hazards was used to compare VTE risk, stratified by age, sex, BMI, disease duration and prescription of oestrogen-containing contraceptives or hormone replacement therapy (HRT).

**Results:**

VTE risk was higher in individuals with RA (*n* = 23 410) than the matched controls (*n* = 93 640): adjusted hazard ratio 1.46 (95% CI 1.36, 1.56). Absolute risk of VTE increased with age and higher BMI. Compared with the controls, however, the relative excess risk of VTE was higher in younger than older individuals: 18–49 years (2.13; 95% CI 1.62, 2.79); 50–69 years (1.57; 95% CI 1.38, 1.78); ≥70 years (1.34; 95% CI 1.14, 1.60) and higher in individuals with normal vs elevated BMI: <25 kg/m^2^ (1.66; 95% CI 1.39, 1.98); 25–30 kg/m^2^ (1.60; 95% CI 1.36, 1.88); >30 kg/m^2^ (1.41; 95% CI 1.19, 1.68). VTE risk remained elevated irrespective of disease duration, was similar between women prescribed vs not prescribed oestrogen-containing contraceptives and higher for women prescribed HRT than those not prescribed HRT.

**Conclusion:**

Individuals with RA are at increased risk of VTE regardless of age, sex, BMI, disease duration and exposure to exogenous oestrogens. This highlights the need to consider VTE risk in all individuals with RA.

Rheumatology key messages

- Individuals with RA are at increased risk of VTE, regardless of age, sex, BMI, disease duration or the use of exogenous oestrogens.
- The relative excess risk of VTE is evident within the first 2 years of diagnosis and persists throughout later stages of the disease.

## Introduction

RA is a chronic, immune-mediated inflammatory disease that is associated with substantial morbidity. Previous studies have shown that individuals with RA are at elevated risk of venous thromboembolism (VTE), including deep venous thrombosis (DVT) and pulmonary embolism (PE), when compared with individuals without RA [1–3]. However, it remains poorly understood the extent to which the excess risk of VTE in individuals with RA is influenced by factors including age, sex, BMI, disease duration and exposure to oestrogen-containing contraceptives or hormone replacement therapy (HRT).

A better understanding of the relative and absolute risk of VTE could help in the tailoring of treatment plans and screening protocols based on an individual’s risk profile. This is of particular importance when individuals with RA are undergoing procedures (e.g. surgery), periods of immobilization (e.g. long-haul flights) or being considered for treatments that might increase their risk of VTE. Following the publication of the ORAL Surveillance trial, concerns were raised about signals for increased VTE incidence among individuals with RA treated with Janus kinase (JAK) inhibitors. This led to regulatory warnings being issued about JAK inhibitor use in patients at increased risk of VTE [4, 5]. To help contextualize any potential risk, it is important to characterize how the inherent risk of VTE varies among individuals with RA, and how this compares to individuals without RA.

Our objective was to explore variation in the risk of VTE by age, sex, BMI, disease duration and the prescription of exogenous oestrogens in a large population-based study of individuals with RA.

## Methods

### Study design and data source

We performed a matched cohort study in adults aged 18 years or older, registered with a UK general practice between 1 January 1999 and 31 December 2018. Data were sourced from the Royal College of General Practitioners Research (RCGP) Research and Surveillance Centre (RSC) database: a representative network of general practices distributed across England, covering a registered population of over 2 million people [6, 7]. The RSC contains pseudonymized demographics and coded information on diagnoses, laboratory tests and prescriptions. Studies utilizing RSC data have been published across a range of chronic diseases [8–10], and have reported on outcomes including VTE [11–13].

### Study population

The study population of interest was adults who had prevalent or incident diagnoses of RA during the study period and no prior history of VTE. RA diagnoses were defined using the algorithm published by Muller *et al.* (itself updated from the original algorithm published by Thomas *et al.* which had a sensitivity of 84% and specificity of 86% in identifying patients as having RA) [14, 15]. Controls were adults without diagnostic codes for RA, matched 4:1 with RA individuals using nearest-neighbour matching with replacement by the following variables: current age (per year), sex, calendar time and years since practice registration. The eligible pool of unexposed individuals comprised of people registered at the index date with no history of RA and at least 1 year of follow-up in RSC, to minimize the risk they had a non-recorded existing RA diagnosis. All individuals with RA and controls were required to have had at least one consultation for any cause during the study period, to account for patients who may be registered with, but no longer attending, a general practice. Individuals who later developed RA were allowed to serve as controls prior to RA development; if matched, these individuals were eligible to contribute to unexposed person-time before their RA diagnosis.

The index date for start of follow-up for exposed individuals began on the latest of: the date of diagnosis indicated by first diagnostic code for RA, 1 January 1999 or 180 days after practice registration. Follow-up for each control started on the index date of their matched case. Follow-up for each individual ended at the earliest of: study end-date, the date an individual was transferred from an included practice, date of death or the date an individual developed the outcome of interest.

### Outcome

The primary outcome was a diagnostic code for VTE (a composite of DVT and PE), using updated diagnostic codelists from a previously published algorithm, with no requirement for concomitant exposure to anticoagulants [16]. In exploratory analyses of VTE risk by disease duration, additional outcomes included DVT and PE events analysed separately.

### Statistical methods

Baseline characteristics were tabulated without inferential statistics for individuals with RA and matched controls. Unadjusted absolute incidence rates for VTE were presented per 1000 person-years (py) of exposure for individuals with RA and for matched controls. The relative excess risk of VTE, comparing RA patients with matched controls, was modelled using Cox proportional hazards (single-failure models), and presented as adjusted hazard ratios (aHR) with 95% CIs. Comparisons of VTE risk between individuals with RA and matched controls were performed for the following subgroups: (1) age category (18–49 years, 50–69 years, ≥70 years); (2) sex (female, male); (3) BMI category (<25 kg/m^2^, 25–29.9 kg/m^2^, ≥30 kg/m^2^); (4) RA disease duration at index date (0–2 years, 2–5 years, 5–10 years and ≥10 years from diagnosis) and (5) use of oestrogen-containing contraceptives or HRT in females with RA and matched female controls. Progesterone-only contraceptives were not included, as these preparations are not associated with VTE [17]. HRT comprised systemic oestrogen-only preparations. Active prescribing of oestrogen-containing contraceptives or HRT was defined as an issued prescription in the 3 months preceding, and/or 1 month after, the index date.

Models were adjusted for covariates selected a priori on the basis of whether they could potentially influence VTE risk [2]: age, sex, ethnicity (categorized as White, Black, Asian, mixed and other ethnicity groups), socioeconomic status (index of multiple deprivation [IMD] quintile), BMI (most recently recorded prior to index date), smoking status (most recently recorded prior to index date), alcohol use (most recently recorded prior to index date), evidence of reduced mobility, thrombophilia, fracture of the lower limb, family history of VTE, hypertension, hyperlipidaemia, type 2 diabetes, peripheral vascular disease, atrial fibrillation, myocardial infarction, stroke, heart failure, chronic kidney disease (CKD) stages 3–5, chronic obstructive pulmonary disease (COPD), chronic liver disease, malignancy, prescriptions for NSAIDs, oral corticosteroids, antiplatelet agents (aspirin or ADP receptor inhibitors), warfarin, direct oral anticoagulants (DOACs), statins, HRT or oestrogen-containing contraceptives, non-biologic immunosuppressant medications and/or biologic therapies recorded in primary care. Immunosuppressant and biologic therapies prescribed solely in secondary care were not captured. Missing data for variables were included in models as an additional category; this method was chosen over multiple imputation, as data were deemed likely to be missing not at random.

Proportional hazards assumptions were checked graphically using Schoenfeld residuals (Supplementary Table S1). Interactions between case and control status and subgroups of age, sex, BMI, disease duration and use of oestrogen-containing contraceptives or HRT were assessed, whereby *P*-values represented the statistical significance of global interaction tests (*F*-tests) comparing models with and without interaction terms. All analyses were performed in R (version 4.4.1; R Foundation for Statistical Computing, Vienna, Austria).

### Ethical approval

Study approval was granted by the Research Committee of the RCGP RSC. The study did not meet the requirements for formal ethics board review, as defined using the NHS Health Research Authority research decision tool (http://www.hra-decisiontools.org.uk/research/).

## Results

A total of 117 050 individuals were included, of whom 23 410 had diagnoses of RA and 93 640 were matched controls. Average follow-up was 8.2 years (S.D., 6.6 years). Sociodemographic and clinical characteristics of individuals with RA were comparable to matched controls (Table 1; Supplementary Fig. S1). The mean age of individuals with RA was 59.0 years (S.D. 15.5), 71.1% were female and mean BMI was 27.1 kg/m^2^ (S.D. 5.6), 63.9% were included in the cohort at or within 2 years of RA diagnosis, 7.8% within 2–5 years of diagnosis, 9.8% within 5–10 years of diagnosis and 18.5% at 10 years or more after diagnosis. Individuals with RA were prescribed NSAIDs more frequently than matched controls (53.4% vs 27.2%), as well as oral corticosteroids (26.7% vs 5.9%) and immunosuppressant medications in a primary care setting (48.2% vs 0.9%).

**Table 1.** Baseline characteristics of individuals with RA and matched controls

	Matched controls	RA cases
	*n* = 93 640	*n* = 23 410
Mean age at entry, years (S.D.)	58.7 (16.2)	59.0 (15.5)
Female sex (%)	65 448 (69.9)	16 634 (71.1)
Index of multiple deprivation quintile		
1	13 089 (14.0)	3477 (14.9)
2	14 340 (15.3)	3728 (15.9)
3	18 254 (19.5)	4739 (20.2)
4	21 938 (23.4)	5326 (22.8)
5	24 062 (25.7)	5651 (24.1)
Missing	1957 (2.1)	489 (2.1)
Ethnicity		
White	66 416 (70.9)	17 119 (73.1)
Asian	3731 (4.1)	1114 (4.8)
Black	1873 (2.2)	403 (1.7)
Mixed	549 (0.6)	150 (0.6)
Other	497 (0.6)	123 (0.5)
Missing	20 574 (22.0)	4501 (19.2)
Duration of RA diagnosis, years		
0–2	NA	14951 (63.9)
2–5	NA	1822 (7.8)
5–10	NA	2305 (9.8)
10+	NA	4332 (18.5)
Calendar year of entry		
1999	13 512 (14.4)	3378 (14.4)
2000–2009	32 828 (35.2)	8207 (35.2)
2010–2019	47 300 (50.5)	11 825 (50.5)
VTE risk factors		
BMI category, kg/m^2^		
<25	32 977 (35.2)	8365 (35.7)
25–29.9	30 858 (33.0)	7735 (33.0)
≥30	21 076 (22.5)	5787 (24.7)
Missing	8729 (9.3)	1523 (6.5)
Smoking status		
Never smoked	40 712 (43.5)	8853 (37.8)
Active smoker	21 055 (22.5)	6063 (25.9)
Ex-smoker	31 121 (33.2)	8385 (35.8)
Not recorded	752 (0.8)	109 (0.5)
Alcohol use		
Within limits	53 726 (57.4)	13 164 (56.2)
Non-drinker	17 733 (18.9)	5404 (23.1)
Over recommended limits	12 037 (12.9)	2767 (11.8)
Alcoholism	1364 (1.5)	340 (1.5)
Not recorded	8780 (9.4)	1735 (7.4)
Reduced mobility	2064 (2.2)	636 (2.7)
Thrombophilia	68 (0.1)	20 (0.1)
Family history of VTE	152 (0.2)	37 (0.2)
History of fracture	6677 (7.1)	1849 (7.9)
Comorbidities		
Hypertension	25 861 (27.6)	6809 (29.1)
Hyperlipidaemia	28 486 (30.4)	6850 (29.3)
Type 2 diabetes mellitus	7201 (7.7)	1993 (8.5)
Peripheral vascular disease	1162 (1.2)	312 (1.3)
Atrial fibrillation	2894 (3.1)	779 (3.3)
Myocardial infarction	2552 (2.7)	767 (3.3)
Stroke	2059 (2.2)	489 (2.1)
Heart failure	1465 (1.6)	429 (1.8)
Chronic kidney disease (stages 3–5)	4551 (4.9)	1226 (5.2)
Chronic obstructive pulmonary disease	3462 (3.7)	1325 (5.7)
Chronic liver disease	509 (0.5)	203 (0.9)
Malignancy	5269 (5.6)	1265 (5.4)
Medication use in primary care		
NSAIDs	25 464 (27.2)	12 501 (53.4)
Corticosteroids	5564 (5.9)	6256 (26.7)
Immunosuppressants	808 (0.9)	11 279 (48.2)
Statins	17 653 (18.9)	4764 (20.4)
Antiplatelet agents	10 695 (11.4)	2784 (11.9)
Warfarin	1805 (1.9)	489 (2.1)
Direct oral anticoagulants	632 (0.7)	193 (0.8)
Hormone replacement therapy	2461 (2.6)	753 (3.2)
Oral contraceptive therapy	2542 (2.7)	546 (2.3)

VTE: venous thromboembolism.

**Figure 1.**
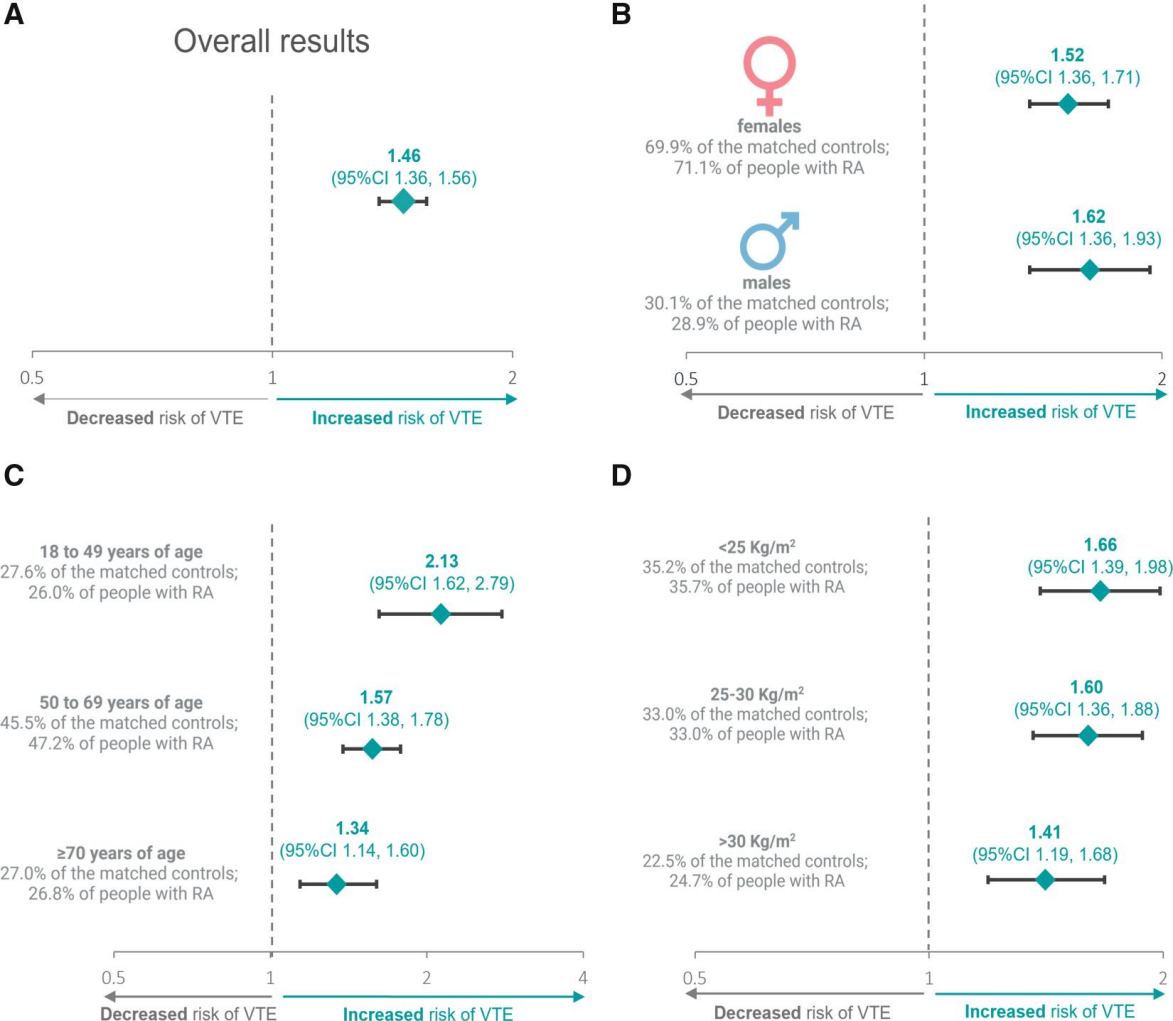
Relative excess risk of venous thromboembolism (VTE) in individuals with RA, compared with matched controls, shown for the full study cohort (Panel A), by sex (Panel B), by age group (Panel C) and by BMI (Panel D). Estimates are from multivariable-adjusted Cox proportional hazards models, reported as adjusted hazard ratios with 95% CIs. Covariates included sociodemographic and clinical characteristics, and established VTE risk factors, as detailed in the methods section

During the study period, there were 845 VTE events in 23 410 individuals with RA (190 220 py of exposure) and 2020 VTE events in 93 640 controls (770 424 py). Unadjusted absolute incidence rates for VTE were higher in people with RA (4.42 events per 1000 py; 95% CI 4.13, 4.73) than matched controls (2.62 per 1000 py; 95% CI 2.52, 2.74) (Table 2; Supplementary Fig. S2). In multivariable-adjusted Cox models, the risk of VTE was higher in individuals with RA than matched controls (adjusted HR 1.46; 95% CI 1.36, 1.56; *P* < 0.001) (Fig. 1A; Table 2).

**Table 2.** Incidence of venous thromboembolism (VTE) in individuals with RA and matched controls

	Population	Number of individuals	Number of VTE events	Unadjusted IR per 1000 person-years (95% CI)	Adjusted HR (95% CI)
Overall					
	Controls	93 640	2020	2.62 (2.52, 2.74)	1.46 (1.36, 1.56) *P* < 0.001
	RA	23 410	845	4.42 (4.13, 4.73)	
Age (years)					
18–49	Controls	25 811	198	0.92 (0.79, 1.05)	2.13 (1.62, 2.79) *P* < 0.001
	RA	6,080	108	2.10 (1.72, 2.53)	
50–69	Controls	42 560	1040	2.65 (2.49, 2.82)	1.57 (1.38, 1.78) *P* < 0.001
	RA	11 048	457	4.56 (4.15, 5.00)	
≥70	Controls	25 269	782	4.83 (4.50, 5.18)	1.34 (1.14, 1.60) *P* < 0.001
	RA	6,282	280	7.12 (6.31, 8.00)	
Sex					
Male	Controls	28 192	605	2.66 (2.46, 2.88)	1.62 (1.36, 1.93) *P* < 0.001
	RA	6,776	255	4.73 (4.17, 5.35)	
Female	Controls	65 448	1415	2.61 (2.47, 2.74)	1.52 (1.36, 1.71) *P* < 0.001
	RA	16 634	590	4.30 (3.96, 4.66)	
BMI (kg/m^2^)					
<25	Controls	32 977	528	1.95 (1.78, 2.12)	1.66 (1.39, 1.98) *P* < 0.001
	RA	8,365	236	3.38 (2.96, 3.84)	
25–29.9	Controls	30 858	700	2.63 (2.43, 2.83)	1.60 (1.36, 1.88) *P* < 0.001
	RA	7,735	296	4.49 (3.99, 5.03)	
≥30	Controls	21 076	662	3.85 (3.56, 4.15)	1.41 (1.19, 1.68) *P* < 0.001
	RA	5,787	262	5.81 (5.13, 6.56)	
Disease duration (years)					
0–2	Controls	59 804	1086	2.58 (2.43, 2.73)	1.49 (1.30, 1.71) *P* < 0.001
	RA	14 951	470	4.49 (4.09, 4.91)	
2–5	Controls	7,288	207	2.65 (2.30, 3.04)	1.56 (1.18, 2.07) *P* < 0.001
	RA	1,822	80	4.15 (3.29, 5.17)	
5–10	Controls	9,220	259	2.59 (2.29, 2.93)	1.61 (1.26, 2.06) *P* < 0.001
	RA	2,305	106	4.26 (3.49, 5.15)	
≥10	Controls	17 328	468	2.73 (2.49, 2.99)	1.63 (1.35, 1.96) *P* < 0.001
	RA	4,332	189	4.49 (3.87, 5.18)	
Oestrogen-containing contraceptive use in females					
Yes	Controls	2,542	11	0.76 (0.38, 1.37)	1.43 (0.60, 3.63), *P* = 0.46
	RA	545	8	2.62 (1.13, 5.16)	
No	Controls	62 906	1404	2.66 (2.52, 2.80)	1.52 (1.36, 1.70) *P* < 0.001
	RA	16 089	582	4.34 (4.00, 4.71)	
Hormone replacement therapy use in females					
Yes	Controls	2,460	25	1.37 (0.88, 2.02)	2.32 (0.92, 5.85), *P* = 0.08
	RA	752	26	4.99 (3.26, 7.31)	
No	Controls	62 988	1390	2.65 (2.51, 2.79)	1.51 (1.35, 1.70) *P* < .001
	RA	15 882	564	4.27 (3.93, 4.64)	

Incidence rates (IRs) are shown, stratified by age, sex, BMI, disease duration and use of oestrogen-containing contraceptives and hormone replacement therapy. Unadjusted absolute IR are shown per 1000 person-years of exposure. Multivariable-adjusted IR, comparing individuals with RA and matched controls, are shown as adjusted hazard ratios (HRs) with 95% CIs.

Absolute incidence rates of VTE were higher in males (4.73 per 1000 py; 95% CI 4.17, 5.35) than females with RA (4.30 per 1000 py; 95% CI 3.96, 4.66) (Table 2). Following multivariable adjustment, the relative excess risk of VTE in individuals with RA, compared with matched controls, was 1.62 (95% CI 1.36, 1.93; *P* < 0.001) for males and 1.52 (95% CI 1.36, 1.71; *P* < 0.001) for females (Fig. 1B). There was no evidence of a significant interaction between sex, the presence or absence of RA and VTE risk (*P* = 0.33).

The absolute incidence of VTE increased with age in individuals with RA and matched controls (Table 2). Compared with the controls, the relative excess risk of VTE was greater in younger people with RA than in older age groups: 18–49 years (aHR 2.13; 95% CI 1.62, 2.79; *P* < 0.001); 50–69 years (aHR 1.57; 95% CI 1.38, 1.78; *P* < 0.001); 70 years or more (aHR 1.34; 95% CI 1.14, 1.60; *P* < 0.001) (Fig. 1C). A significant interaction was evident between age group, the presence or absence of RA and VTE risk (*P* = .01).

Absolute incidence rates of VTE increased with higher BMI in individuals with RA and matched controls (Table 2). Compared with the controls, the relative excess risk of VTE was greater in individuals with a BMI <25 kg/m^2^ (aHR 1.66; 95% CI 1.39, 1.98; *P* < 0.001) or 25–30 kg/m^2^ (aHR 1.60; 95% CI 1.36, 1.88; *P* < 0.001), than those with a BMI >30 kg/m^2^ (aHR 1.41; 95% CI 1.19, 1.68; *P* < 0.001) (Fig. 1D). There was no evidence of a significant interaction between BMI, the presence or absence of RA and VTE risk (*P* = 0.40).

**Figure 2.**
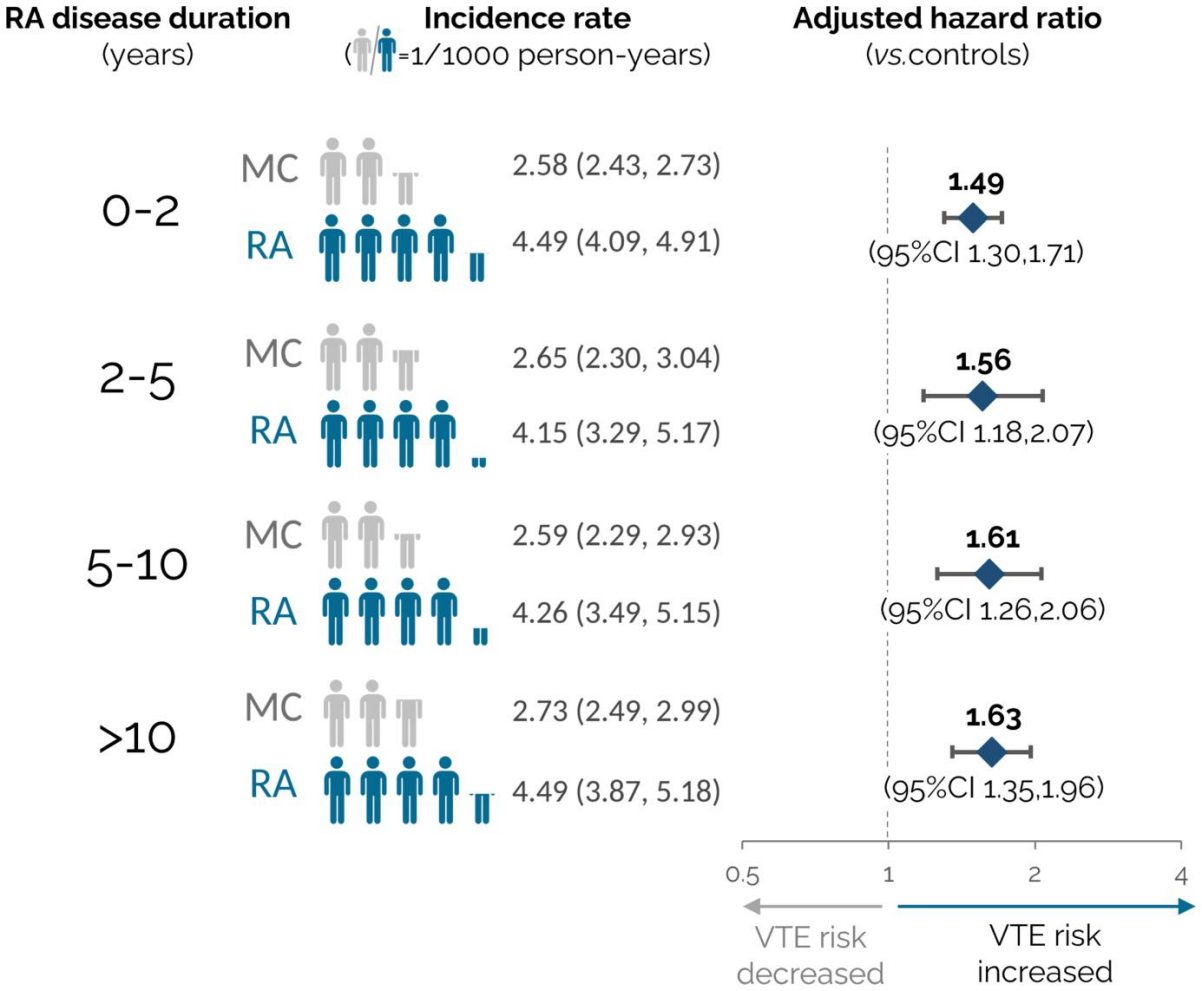
Incidence of venous thromboembolism (VTE) in people with RA, relative to matched controls, by duration of disease. Unadjusted absolute incidence rates are presented per 1000 person-years of exposure, in addition to multivariable-adjusted hazard ratios with 95% CIs comparing VTE risk in individuals with RA and matched controls. Covariates included sociodemographic and clinical characteristics, and established VTE risk factors, as detailed in the Methods section

Absolute incidence rates of VTE were consistently elevated in people with RA regardless of disease duration (Fig. 2; Table 2). The adjusted HR for VTE, relative to controls, was 1.49 (95% CI 1.30, 1.71; *P* < 0.001) for individuals with a disease duration of 0–2 years; 1.56 (95% CI 1.18, 2.07; *P* < 0.001) for a disease duration of 2–5 years; 1.61 (95% CI 1.26, 2.06; *P* < 0.001) for a disease duration of 5–10 years and 1.63 (95% CI 1.35, 1.96; *P* < 0.001) for a disease duration of >10 years. In exploratory analyses, comparable findings were observed when analysing DVT and PE separately (Supplementary Fig. S3 and Supplementary Table S2). There was no evidence of interaction between disease duration and VTE risk in RA patients, relative to the controls (*P* = 0.80).

**Figure 3.**
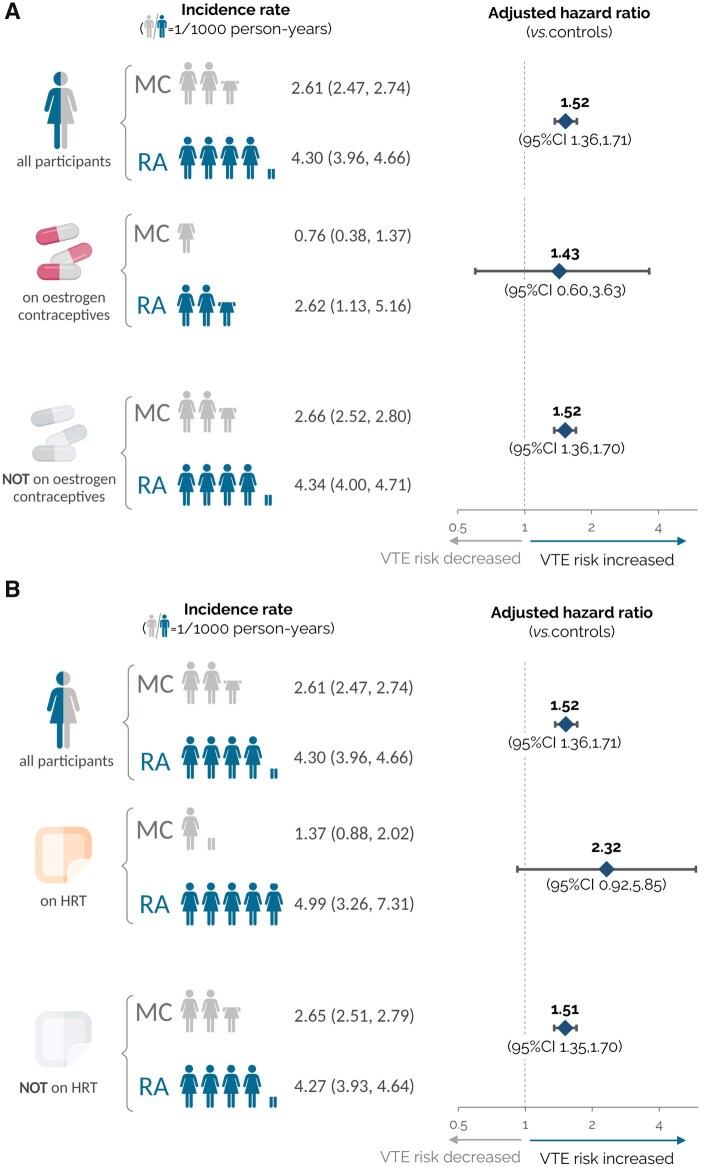
Incidence of venous thromboembolism (VTE) in female individuals with RA, relative to matched female controls, comparing individuals prescribed vs not prescribed oestrogen-containing contraceptives (Panel A) or hormone replacement therapy (HRT) (Panel B). Unadjusted absolute incidence rates (IR) for VTE are shown per 1000 person-years of exposure, in addition to multivariable-adjusted hazard ratios with 95% CIs comparing VTE risk in female individuals with RA and matched female controls. Covariates included sociodemographic and clinical characteristics, and established VTE risk factors, as detailed in the Methods section

When restricted to females with RA (*n* = 16 634) and matched female controls (*n* = 65 448), 3.3% and 3.9%, respectively, were prescribed oestrogen-containing contraceptives at the index date, while 4.5% and 3.8%, respectively, were prescribed HRT (Supplementary Table S3). The relative excess risk of VTE in females with RA, compared with the controls, was similar between individuals prescribed oestrogen-containing contraceptives (aHR 1.43; 95% CI 0.60, 3.63; *P* = 0.46) and those not prescribed oestrogen-containing contraceptives (aHR 1.52; 95% CI 1.36, 1.70; *P* < 0.001) (Table 2; Fig. 3A). The relative excess risk of VTE in female individuals with RA prescribed HRT, compared with matched female controls prescribed HRT, was 2.32 (95% CI 0.92, 5.85; *P* = 0.08); for individuals not prescribed HRT, the adjusted HR was 1.51 (95% CI 1.35, 1.70; *P* < 0.001) (Table 2; Fig. 3B). A significant interaction was evident between HRT use, the presence or absence of RA and VTE risk (*P* = 0.01), but not for oestrogen-containing contraceptives use and VTE risk (*P* = 0.24).

## Discussion

In this large population-based cohort study, we showed that individuals with RA are at an elevated risk of VTE, regardless of age, sex, BMI, disease duration or the use of exogenous oestrogens. The elevated risk of VTE was evident within the first 2 years of diagnosis and persisted throughout later stages of the disease. While the absolute risk of VTE increased with age and BMI, the relative excess risk of VTE was greatest in individuals aged under 50 years and in those without obesity.

Our finding of an elevated risk of VTE in individuals with RA is in keeping with several previous studies [1–3, 18–23], and is thought to be driven by numerous factors, including the systemic inflammatory state, reduced mobility and treatments such as corticosteroids [24, 25]. We demonstrated that the elevated risk of VTE was present regardless of disease duration, consistent with findings from an earlier population-based study [20]. In our analyses, we were able to account for multiple potential confounders of VTE risk, including comorbid health conditions, medication use, reduced mobility and fracture history. After adjusting for these factors, individuals with RA remained 50% more likely to experience VTE than those unaffected by RA. This risk was evident not only in individuals with longstanding RA but also in those with very early disease, emphasizing the need for vigilant monitoring and assessment of VTE risk in all patients with RA.

These data provide important context for clinicians and patients who are considering procedures or treatments that might increase VTE risk. This has been a topic of extensive debate following the publication of the ORAL Surveillance trial and observational data reporting signals for increased VTE incidence associated with JAK inhibitor use in individuals with RA, relative to biologic DMARDs [4, 5]. In the presence of safety warnings being issued on JAK inhibitor use by regulatory bodies in the UK, Europe and the USA, our findings can help to contextualize the risk of VTE in individuals with RA, highlighting the important distinction between absolute and relative risk of VTE.

We showed that individuals with RA aged 70 years or older, and those who are obese, are far more likely to experience VTE events than younger individuals or those with normal BMI. Individuals with RA who are aged under 50 years and those with normal BMI remain significantly more likely to experience VTE than individuals without RA; however, their absolute risk of VTE is much lower overall. In these circumstances, when considering treatments or procedures that might increase the risk of VTE, it is important to explore additional factors that could influence risk–benefit decisions; for example, a previous history of VTE, malignancy, chronic kidney disease, active smoking or corticosteroid use [2, 26].

This is the first comprehensive analysis of VTE risk associated with exposure to exogenous oestrogens in individuals with RA. After adjusting for differences in age and other key factors, we showed that the relative excess risk of VTE was comparable between females prescribed oestrogen-containing contraceptives and those not prescribed oestrogen-containing contraceptives, whilst the risk of VTE was greater in those prescribed HRT than those not prescribed HRT. Both estimates had wide confidence intervals, reflecting the relatively small number of VTE events in the sub-populations receiving exogenous oestrogens. While further studies are needed, our findings suggest that female individuals with RA should be considered at increased risk of VTE relative to RA-unaffected individuals, irrespective of whether they are prescribed exogenous oestrogens or not.

The strengths of our study include its large sample size and long duration of follow-up, utilizing an established dataset that is representative of England’s population [27]. The exposures and outcomes in our study were identified using previously validated algorithms, and we were able to adjust for multiple potential confounders of VTE risk.

Our study also had limitations. This study utilized routinely collected primary care data from the UK, and therefore the findings are not necessarily generalizable to other countries or healthcare systems. There is the potential for unmeasured confounding and diagnostic misclassification inherent to studies utilizing routinely collected health data. Data on disease activity and inflammation burden were not available, and data on immunosuppressant medications were limited to those prescribed in primary care. Missing data on secondary care-prescribed medicines and disease activity are of particular relevance to studies evaluating VTE outcomes in inflammatory disorders, given their potential roles as pathway variables in the development and/or mitigation of VTE risk [22]. We could not adjust for these factors in our analyses, which must be considered when interpreting the results of our study. The number of VTE events in individuals with RA who were prescribed oestrogen-based therapies was low, which resulted in estimates with wide confidence intervals. Additionally, individuals were assumed to be taking oestrogen-based therapies if they had been prescribed them at the index date; however, this would not account for individuals who stopped or started these therapies at a later date. Some variables had missing data, including BMI and ethnicity, for which we utilized the missing indicator variable method as data were deemed likely to be missing not at random, meaning multiple imputation may lack validity [28].

In conclusion, individuals with RA are at increased risk of VTE regardless of age, sex, BMI, disease duration and exposure to exogenous oestrogens. The excess risk of VTE is apparent within 2 years of disease onset, and is relatively more marked among younger individuals with RA when compared with individuals without RA. This has important clinical implications, and highlights the need to consider VTE risk in all patients diagnosed with RA.

## Supplementary Material

keaf430_Supplementary_Data

## Data Availability

No additional data are available from the authors, due to the confidential patient data utilized in these analyses; however, applications to analyse data in the Royal College of General Practitioners (RCGP) Research and Surveillance Centre (RSC) database can be submitted to the Research Committee of the RCGP RSC.
