# Supplementary material for: Risk of venous thromboembolism in people with RA: a population-based study in the UK
Source: Rheumatology (Oxford). 2025 Aug 7;64(12):6224–32. doi: 10.1093/rheumatology/keaf430 (PMC12671859; doi:10.1093/rheumatology/keaf430)
Supplement: keaf430_Supplementary_Data [file keaf430_supplementary_data.docx]

**
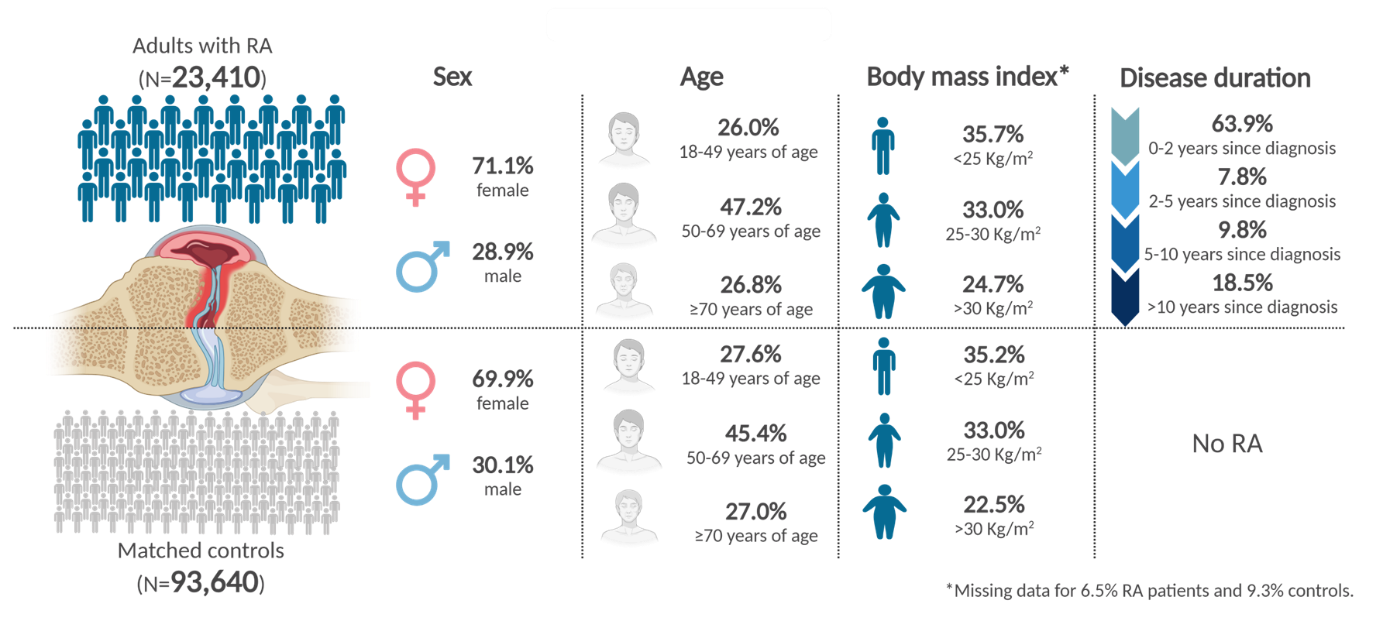
Supplementary Figure S1**: Distribution of age, sex, body mass index, and disease duration for individuals with RA and matched controls.

**
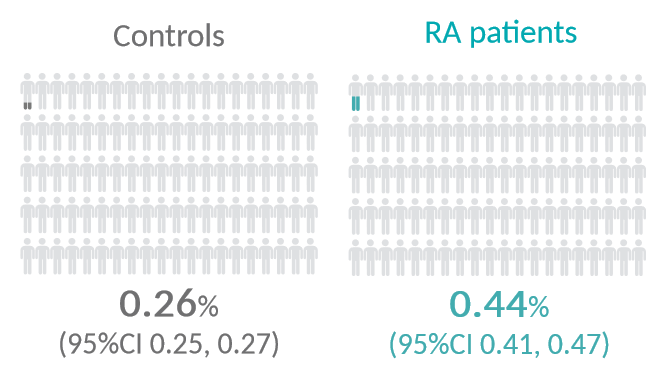
Supplementary Figure S2:** Absolute incidence of venous thromboembolism (VTE) in people with RA, relative to matched controls.

The proportion of individuals with rheumatoid arthritis (RA) and matched controls who experienced a VTE event per year is depicted graphically, and represent unadjusted estimates of absolute incidence with 95% confidence intervals (CI).


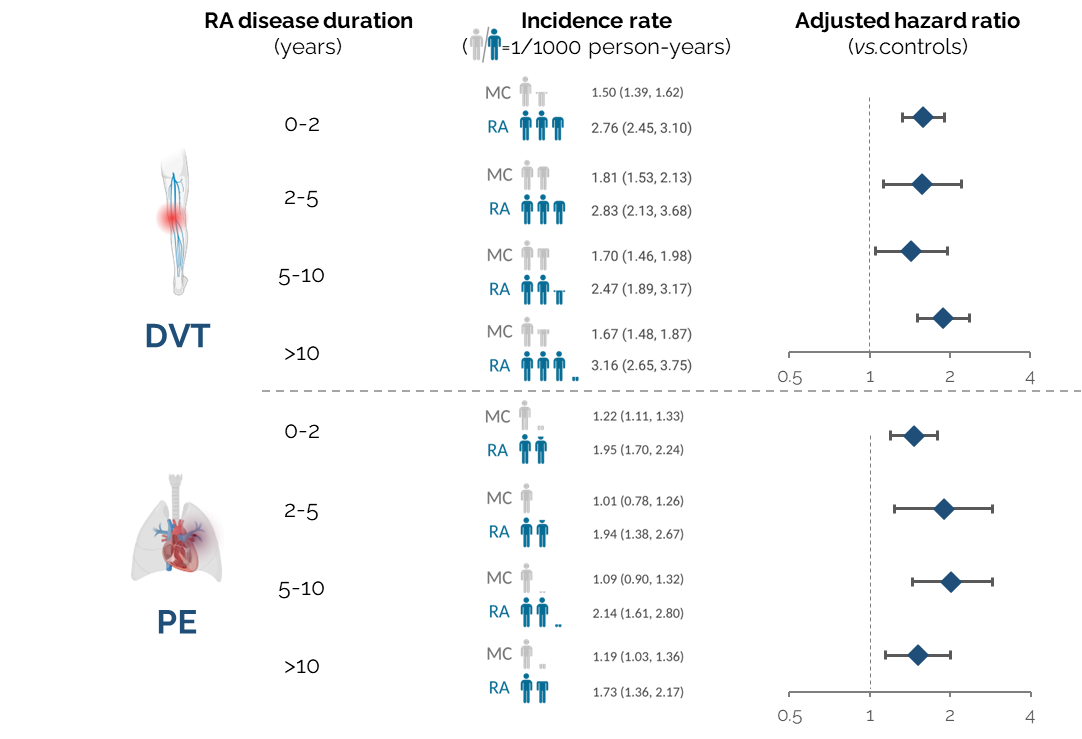
**Supplementary Figure S3:** Incidence of DVT and PE, stratified by disease duration, in individuals with RA and matched controls.

Unadjusted absolute incidence rates (IR) for deep venous thrombosis (DVT) and pulmonary embolism (PE) are shown per 1,000 person-years of exposure, in addition to multivariable-adjusted hazard ratios with 95% confidence intervals (CI), comparing VTE risk in individuals with RA and matched controls. Covariates included sociodemographic and clinical characteristics, and established VTE risk factors, as detailed in the methods section.

**Supplementary Table S1**. Proportional hazards assumption tests.

| **Subgroup** | **P-value** |
| --- | --- |
| Overall | 0.83 |
| Age 18-49 years | 0.31 |
| Age 50-69 years | 0.52 |
| Age ≥70 years | 0.61 |
| Males | 0.78 |
| Females | 0.12 |
| BMI <25 kg/m^2^ | 0.62 |
| BMI 25-29.9 kg/m^2^ | 0.95 |
| BMI ≥30 kg/m^2^ | 0.98 |
| Disease duration 0-2 years | 0.77 |
| Disease duration 2-5 years | 0.29 |
| Disease duration 5-10 years | 0.80 |
| Disease duration 10+ years | 0.39 |
| Females with oestrogen-containing contraceptive use | 0.54 |
| Females without oestrogen-containing contraceptive use | 0.60 |
| Females with hormone replacement therapy | 0.77 |
| Females without hormone replacement therapy | 0.56 |

Estimates were obtained from multivariable-adjusted Cox proportional hazards models for each subgroup.

**Supplementary** **Table S2:** Incidence of DVT and PE in individuals with RA, compared with matched controls.

| **Disease duration** | **Population** | **Number of individuals** | **Number of VTE events** | **Unadjusted IR per 1,000 person-years (95% CI)** | **Adjusted HR**  **(95% CI)** |
| --- | --- | --- | --- | --- | --- |
| **Pulmonary embolism** | | | | | |
| 0-2 years | Controls | 59,804 | 518 | 1.22 (1.11, 1.33) | 1.46 (1.20, 1.79)  p<0.001 |
|  | RA | 14,951 | 207 | 1.95 (1.70, 2.24) |  |
| 2-5 years | Controls | 7,288 | 80 | 1.01 (0.78, 1.26) | 1.89 (1.24, 2.88)  p<0.001 |
|  | RA | 1,822 | 38 | 1.94 (1.38, 2.67) |  |
| 5-10 years | Controls | 9,220 | 111 | 1.09 (0.90, 1.32) | 2.02 (1.44, 2.87)  p<0.001 |
|  | RA | 2,305 | 54 | 2.14 (1.61, 2.80) |  |
| ≥10 years | Controls | 17,328 | 207 | 1.19 (1.03, 1.36) | 1.51 (1.14, 2.00)  p<0.001 |
|  | RA | 4,332 | 74 | 1.73 (1.36, 2.17) |  |
| **Deep vein thrombosis** | | | | | |
| 0-2 years | Controls | 59,804 | 638 | 1.50 (1.39, 1.62) | 1.59 (1.33, 1.90)  p<0.001 |
|  | RA | 14,951 | 291 | 2.76 (2.45, 3.10) |  |
| 2-5 years | Controls | 7,288 | 143 | 1.81 (1.53, 2.13) | 1.57 (1.13, 2.21)  p<0.001 |
|  | RA | 1,822 | 55 | 2.83 (2.13, 3.68) |  |
| 5-10 years | Controls | 9,220 | 172 | 1.70 (1.46, 1.96) | 1.43 (1.05, 1.96)  p<0.001 |
|  | RA | 2,305 | 62 | 2.47 (1.90, 3.17) |  |
| ≥10 years | Controls | 17,328 | 289 | 1.67 (1.48, 1.87) | 1.89 (1.51, 2.36)  p<0.001 |
|  | RA | 4,332 | 134 | 3.16 (2.65, 3.75) |  |

Unadjusted absolute incidence rates (IR) are shown per 1,000 person-years of exposure. Multivariable-adjusted incidence rates, comparing individuals with RA and matched controls, are shown as adjusted hazard ratios. BMI, body-mass index; CI, confidence interval; HR, hazard ratio; RA, rheumatoid arthritis; HRT, hormone replacement therapy.

**Supplementary Table S3**. Baseline characteristics of female individuals with RA and matched female controls.

|  | **Matched control females**  **n=65,448** | **RA cases females**  **n=16,634** |
| --- | --- | --- |
| Mean age at entry, years (SD) | 58.3 (16.5) | 58.5 (15.9) |
| Index of multiple deprivation quintile |  |  |
| 1 | 9326 (14.2) | 2533 (15.2) |
| 2 | 10233 (15.6) | 2651 (15.9) |
| 3 | 12581 (19.2) | 3339 (20.1) |
| 4 | 15208 (23.2) | 3716 (22.3) |
| 5 | 16756 (25.6) | 4055 (24.4) |
| Missing | 1344 (2.1) | 340 (2.0) |
| Ethnicity |  |  |
| White | 46397 (70.9) | 12037 (73.1) |
| Asian | 2707 (4.1) | 871 (5.2) |
| Black | 1430 (2.2) | 328 (2.0) |
| Mixed | 417 (0.6) | 121 (0.7) |
| Other | 368 (0.6) | 101 (0.6) |
| Missing | 14129 (21.6) | 3176 (19.2) |
| Duration of RA diagnosis, years |  |  |
| 0-2 | NA | 10365 (62.3) |
| 2-5 | NA | 1307 (7.9) |
| 5-10 | NA | 1700 (10.2) |
| 10+ | NA | 3262 (19.6) |
| **VTE risk factors** |  |  |
| BMI category, kg/m^2^ |  |  |
| <25 | 25070 (38.3) | 6312 (37.9) |
| 25-29.9 | 19537 (29.9) | 4966 (29.9) |
| ≥30 | 15024 (23.0) | 4270 (25.7) |
| Missing | 5817 (8.9) | 1086 (6.5) |
| Smoking status |  |  |
| Never smoked | 30978 (47.3) | 7074 (42.5) |
| Active smoker | 13872 (21.2) | 4051 (24.4) |
| Ex-smoker | 20123 (30.7) | 5436 (32.7) |
| Not recorded | 475 (0.7) | 73 (0.4) |
| Alcohol use |  |  |
| Within limits | 38989 (59.6) | 9627 (57.9) |
| Non-drinker | 14361 (21.9) | 4413 (26.5) |
| Over recommended limits | 5385 (8.2) | 1216 (7.3) |
| Alcoholism | 612 (0.9) | 166 (1.0) |
| Not recorded | 6101 (9.3) | 1212 (7.3) |
| Reduced mobility | 1509 (2.3) | 477 (2.9) |
| Thrombophilia | 58 (0.1) | 15 (0.1) |
| Family history of VTE | 137 (0.2) | 33 (0.2) |
| History of fracture | 4477 (6.8) | 1293 (7.8) |
| **Comorbidities** |  |  |
| Hypertension | 17577 (26.9) | 4672 (28.1) |
| Hyperlipidaemia | 19512 (29.8) | 4696 (28.2) |
| Type 2 diabetes mellitus | 4319 (6.6) | 1234 (7.4) |
| Peripheral vascular disease | 593 (0.9) | 168 (1.0) |
| Atrial fibrillation | 1621 (2.5) | 438 (2.6) |
| Myocardial infarction | 1071 (1.6) | 345 (2.1) |
| Stroke | 1226 (1.9) | 313 (1.9) |
| Heart failure | 846 (1.3) | 243 (1.5) |
| Chronic kidney disease (stages 3-5) | 3211 (4.9) | 908 (5.5) |
| Chronic obstructive pulmonary disease | 2096 (3.2) | 817 (4.9) |
| Chronic liver disease | 311 (0.5) | 142 (0.9) |
| Malignancy | 3649 (5.6) | 868 (5.2) |
| **Medication use in primary care** |  |  |
| NSAIDs | 17586 (26.9) | 8682 (52.2) |
| Corticosteroids | 4127 (6.3) | 4266 (25.6) |
| Immunosuppressants | 610 (0.9) | 7897 (47.5) |
| Statins | 10552 (16.1) | 2899 (17.4) |
| Antiplatelet agents | 6364 (9.7) | 1681 (10.1) |
| Warfarin | 958 (1.5) | 272 (1.6) |
| Direct oral anticoagulants | 381 (0.6) | 109 (0.7) |
| Hormone replacement therapy | 2460 (3.8) | 752 (4.5) |
| Oral contraceptive therapy | 2542 (3.9) | 545 (3.3) |

BMI: body mass index; NSAID: non-steroidal anti-inflammatory drug; RA: Rheumatoid arthritis; SD: standard deviation; VTE: venous thromboembolism.
